# Screen exposure patterns and their associations with obesity and sleep among preschool children in a resource-limited city in eastern China: a multicenter cross-sectional study protocol

**DOI:** 10.3389/fpubh.2026.1901399

**Published:** 2026-09-17

**Authors:** Mengmeng Zhang, Dandan Shi, Cuiling Gao, Yang Wang, Yuan Fan, Bo Wang

**Affiliations:** 1 Department of Nursing, Suqian First Hospital, Suqian, China; 2 Department of Pediatrics, Suqian First Hospital, Suqian, China; 3 Department of Pediatrics, The Affiliated Huaihai Hospital of Xuzhou Medical University, Xuzhou, China

**Keywords:** cross-sectional study, obesity, preschool children, screen exposure, sleep

## Abstract

**Introduction:**

Electronic screen exposure is increasingly common among preschool children and has been associated with sleep-related outcomes and overweight/obesity. However, evidence from resource-limited areas in eastern China remains limited, particularly regarding multidimensional screen-use patterns. This study aims to describe the prevalence and patterns of screen exposure among preschool children, primarily to examine the association between excessive screen exposure and overweight/obesity, and secondarily to examine associations with sleep-related indicators.

**Methods:**

This kindergarten-based, multicenter analytical cross-sectional study will be conducted in Suqian, a resource-limited city in eastern China. Preschool children aged 3–6 years and their primary caregivers will be recruited using stratified multistage random sampling. Information on screen exposure, child and family characteristics, family media environment, and sleep-related indicators will be collected using a structured questionnaire completed by the child’s primary caregiver. Children’s height and weight will be measured by trained kindergarten staff using standardized procedures. Excessive screen exposure will be defined as average daily screen time >1 h/day, with ≤1 h/day used as the reference category. Overweight/obesity will be the primary outcome. Survey-weighted Poisson regression with a log link and robust variance estimation will be used to estimate prevalence ratios and 95% confidence intervals for the primary association, accounting for the stratified multistage cluster sampling design and survey weights. Associations between screen exposure and individual sleep-related indicators will be examined separately as secondary analyses using survey-weighted ordinal logistic regression models.

**Discussion:**

This study will provide context-specific epidemiological evidence on screen exposure patterns among preschool children in a resource-limited setting in eastern China. By examining multidimensional screen-use behaviors and their association with overweight/obesity, while also assessing sleep-related indicators as secondary outcomes, the study may inform future health promotion strategies and provide a basis for longitudinal research on screen exposure and child health.

**Clinical trial registration:**

https://www.chictr.org.cn; identifier ChiCTR2600126288.

## 1 Introduction

In the context of increasing digitalization, electronic screens have become widely integrated into the daily lives of preschool children (1, 2). The World Health Organization (WHO) and relevant expert recommendations suggest that daily screen exposure for preschool children should be limited to no more than 1 h per day (3, 4). However, excessive screen exposure remains common worldwide (5). Previous studies suggest that screen use among preschool children is characterized by earlier age at first exposure, diverse usage patterns, and insufficient family rules (6–8). These features may be associated with unfavorable health-related outcomes in early childhood. Therefore, it is important to systematically assess the prevalence and patterns of screen exposure in preschool children. This may help identify unfavorable screen-use behaviors and inform future public health strategies.

Previous studies have shown that screen exposure is associated with various adverse health outcomes in preschool children, with sleep and obesity receiving particular attention (7, 8). On the one hand, longer screen time and unfavorable usage patterns, such as screen use before bedtime and background television exposure, may be associated with sleep problems. These problems include shorter sleep duration, delayed sleep onset, and daytime sleepiness (9–11). On the other hand, excessive screen exposure has been associated with a higher prevalence of overweight and obesity. Potential mechanisms underlying this association include increased sedentary behavior, reduced outdoor activity, and changes in eating habits (12, 13). In addition, insufficient sleep has also been associated with overweight and obesity in children (14). This may further complicate the associations between screen exposure and child health outcomes. Taken together, screen exposure is an important behavioral exposure associated with multiple aspects of health and development in preschool children.

Although research on screen exposure in children has increased in recent years, the existing evidence still has clear limitations. In China, most studies have been conducted in economically developed cities such as Beijing, Shanghai, and Chengdu (6, 8, 15). Evidence on preschool children in resource-limited third- and fourth-tier cities in eastern China remains limited. Compared with children from families with higher socioeconomic status, those living in resource-limited areas may be more likely to experience insufficient caregiving resources, weak family media management, and reliance on “digital nanny” practices. These conditions may be associated with greater screen exposure and less favorable screen-use patterns (10, 16). In addition, previous studies have mainly focused on total screen time. Less attention has been given to specific usage patterns, such as background television exposure, screen use before bedtime or during meals, parental co-viewing, and family rules, as well as their associations with child health outcomes (5, 17). At present, evidence for populations with specific geographic and socioeconomic characteristics remains fragmented. This limits the ability to develop targeted and effective intervention strategies for child health.

Based on these considerations, we will conduct a kindergarten-based, multicenter analytical cross-sectional study in Suqian, a resource-limited city in eastern China. The study will first describe the prevalence and patterns of electronic screen exposure among preschool children aged 3–6 years. In the association analyses, screen exposure will be prespecified as the primary exposure, and overweight/obesity as the primary outcome. The primary objective is to evaluate the association between excessive screen exposure and overweight/obesity. We hypothesize that children with excessive screen exposure will have a higher prevalence of overweight/obesity than those whose screen exposure is within the recommended limit. Secondary objectives are to examine the associations between screen exposure characteristics and sleep-related indicators, and to explore how child, family, and family media environment characteristics are associated with screen exposure. This study is expected to provide local epidemiological evidence on screen use and weight-related health among preschool children in resource-limited settings and to inform future longitudinal research and health promotion strategies.

## 2 Methods

### 2.1 Study design

This study is a kindergarten-based, multicenter analytical cross-sectional study to be conducted in Suqian, a resource-limited city in eastern China. The study population will include preschool children aged 3–6 years and their primary caregivers. The study will describe the prevalence and patterns of electronic screen exposure among preschool children. In the association analyses, screen exposure will be prespecified as the primary exposure, and overweight/obesity as the primary outcome. The primary analysis will evaluate the association between excessive screen exposure and overweight/obesity. Associations between screen exposure and sleep-related indicators, as well as associations of child, family, and family media environment characteristics with screen exposure, will be examined as secondary analyses. Information on screen exposure, family characteristics, and sleep-related indicators will be collected using a structured questionnaire completed by the child’s primary caregiver. Before questionnaire completion, the child’s height and weight will be measured by trained kindergarten staff using standardized procedures. The measurement results will be provided individually to the caregiver, who will enter these values into the electronic questionnaire. This is an observational study and does not involve any intervention. The design, conduct, and reporting of the study will follow the Strengthening the Reporting of Observational Studies in Epidemiology (STROBE) statement (18).

### 2.2 Participants

This study will recruit preschool children aged 3–6 years and their primary caregivers from participating kindergartens in Suqian. Special education schools and rehabilitation or education institutions for children with special needs will not be included in the sampling frame. A primary caregiver is defined as a parent, grandparent, or other caregiver who has lived with the child during the past month and is primarily involved in the child’s daily care.

Inclusion criteria:

1. Children aged 3–6 years who are enrolled in the selected kindergartens in Suqian;
2. The questionnaire is completed by the child’s primary caregiver;
3. The primary caregiver is able to understand the questionnaire and provide information based on the child’s actual situation during the past month;
4. The primary caregiver provides informed consent and voluntarily agrees to participate in the study.

Exclusion criteria:

1. The caregiver reports that the child has a previously confirmed diagnosis from a medical institution of a visual impairment that substantially affects conventional electronic screen use, or a neurodevelopmental disorder that may substantially alter screen-use patterns, such as autism spectrum disorder or severe intellectual disability;
2. The respondent is not the child’s primary caregiver.

For some children with neurodevelopmental disorders or visual impairments, screen use may involve assistive communication, special education, or rehabilitation activities and may therefore differ substantially from the routine screen exposure examined in this study. In addition, the underlying neurodevelopmental condition and its treatment, including medications that may affect sleep, appetite, activity level, or body weight, may independently influence the health outcomes of interest and introduce substantial residual confounding. Because the sampling frame is limited to mainstream kindergartens and does not include special education or rehabilitation institutions, the number of such children in the study population is expected to be small. This would also limit the feasibility of reliable stratified analyses or statistical adjustment according to disease type, severity, or treatment status.

### 2.3 Sample size calculation

The sample size was estimated based on the primary study hypothesis, which is to evaluate the association between excessive screen exposure and overweight/obesity among preschool children. A previous study of 971 Chinese preschool children aged 3–6 years reported an overweight/obesity prevalence of 11.8% (19). Therefore, an overall prevalence of approximately 12% was assumed for the sample size calculation. Another study involving 4,985 Chinese preschool children aged 3–6 years found that 34.8% had daily screen time exceeding 1 h (8). Accordingly, the proportion of children with excessive screen exposure was assumed to be approximately 35%.

A systematic review and meta-analysis of screen use and health outcomes among infants, toddlers, and preschool children reported that children with daily screen time >1 h had higher odds of overweight/obesity than those with screen time ≤1 h, with a pooled odds ratio of 1.988 (95% confidence interval: 1.445-2.735) (20). This exposure classification is consistent with that used for the primary exposure in the present study. Based on an overall overweight/obesity prevalence of 12%, an excessive screen exposure prevalence of 35%, and the pooled effect estimate described above, the expected prevalence of overweight/obesity was estimated to be approximately 9.3% among children with screen time ≤1 h/day and 17.0% among those with screen time >1 h/day. With a two-sided significance level of 0.05, a statistical power of 80%, and an expected group allocation ratio of approximately 65:35, a two-sample comparison of independent proportions indicated that approximately 660 children would be required under simple random sampling.

Because the study uses cluster sampling at the kindergarten level, the sample size was further adjusted for within-kindergarten correlation. A methodological review of 246 school-based cluster randomized trials reported a median school-level intracluster correlation coefficient (ICC) of 0.031 across health outcomes. Among 17 studies reporting body mass index outcomes, the median ICC was 0.021, with an interquartile range of 0.015–0.040 (21). An ICC of 0.02 was therefore adopted for study planning.

In each selected kindergarten, one class will be randomly selected from each of the three grades. Each class is expected to include approximately 30–40 children. Thus, approximately 90–120 children will be invited per kindergarten. For a conservative sample size calculation, the lower estimate of 30 children per class was used. After allowing for an overall reduction of approximately 20% due to non-response, invalid questionnaires, or missing data on the primary exposure or primary outcome, approximately 72 children per kindergarten are expected to be available for the primary analysis. The corresponding design effect was calculated as 1+(m−1)×ICC , yielding 1+(72−1)×0.02=2.42 .

Based on this conservative scenario, 24 kindergartens would yield an estimated 2,160 children eligible for invitation (24×3×30) . After allowing for the anticipated 20% reduction, approximately 1,728 children are expected to remain available for the primary analysis. After accounting for the design effect, this corresponds to an effective sample size of approximately 714 (1,728/2.42) , which exceeds the approximately 660 children required under simple random sampling. Therefore, the study plans to include approximately 24 kindergartens, with one class randomly selected from each grade in each kindergarten. This sampling strategy is expected to provide adequate statistical power for the primary association analysis.

### 2.4 Sampling method and survey implementation

#### 2.4.1 Sampling method

This study will use a stratified multistage random sampling method. The five administrative areas of Suqian—Sucheng District, Suyu District, Shuyang County, Sihong County, and Siyang County—will serve as the primary stratification variables. Before formal sampling, the research team will obtain an updated sampling frame of eligible kindergartens from the local education authorities. The sampling frame will include information on administrative area, kindergarten type (public or private), the number of classes in each grade, and the number of enrolled children aged 3–6 years, where available.

Approximately 24 kindergartens will be selected. The number of kindergartens allocated to each administrative area will be determined in proportion to the number of enrolled children aged 3–6 years in that area relative to the citywide target population. Within each administrative area, kindergartens will be further stratified by type (public or private). The number of kindergartens selected from each type will be allocated in proportion to the number of enrolled children aged 3–6 years, based on the sampling-frame data. Within each stratum, the required number of kindergartens will be randomly selected using computer-generated random numbers.

Within each selected kindergarten, one class will be randomly selected from each of the three grades according to the kindergarten’s actual grade structure. Classes will be selected using computer-generated random numbers. The primary caregivers of all eligible children in the selected classes will be invited to participate. Each class is expected to include approximately 30–40 children; therefore, approximately 90–120 children per kindergarten are expected to be eligible for invitation. The actual number of participants contributed by each kindergarten may vary according to class size and response rate, and equal sample sizes across kindergartens will not be imposed.

After formal sampling has been completed, the overall sampling probability for each child will be calculated based on the actual selection probabilities at the sampling-stratum, kindergarten, and class levels. The base survey weight will be defined as the inverse of the overall sampling probability and will be adjusted for non-response within the relevant sampling strata. Where appropriate, and if corresponding population-level information is available from the local education authorities, the weights will be further calibrated according to variables such as administrative area, kindergarten type, and grade. Details of the weighted analyses will be specified in the statistical analysis section.

#### 2.4.2 Survey implementation

The study will primarily use an electronic questionnaire developed using Wenjuanxing, a WeChat-based survey platform. The first page of the questionnaire will provide information on the study purpose, survey content, participation procedures, privacy protection, voluntary participation, and the right to withdraw. Primary caregivers will be required to read this information and select “I agree to participate in this study” before proceeding to the questionnaire. Those who do not provide consent will exit the survey. The questionnaire is expected to take approximately 8–10 min to complete.

The questionnaire link or QR code will be distributed to parent WeChat groups of the selected classes with the assistance of kindergarten teachers. The questionnaire will be completed by the child’s primary caregiver based on the child’s actual situation during the past month. For caregivers who are unable to complete the electronic questionnaire independently because of limited digital skills, internet access, or device availability, assistance will be provided as needed. Trained class teachers or research staff may assist with electronic data entry based strictly on the responses provided by the primary caregiver. They will not answer questions on behalf of the caregiver or interpret, prompt, or modify the caregiver’s responses. For relatively sensitive items, such as household economic information, caregivers will be encouraged to review the questions and provide their responses themselves whenever possible to protect privacy and minimize social desirability bias.

Before questionnaire completion, eligible children whose primary caregivers have provided informed consent will have their height and weight measured by trained kindergarten staff using standardized procedures. The measurement results will be communicated individually to the primary caregiver, who will enter the values obtained from the current measurements into the corresponding questionnaire items. Detailed anthropometric measurement procedures and quality-control measures will be described in the subsequent section.

Appropriate duplicate-submission restrictions available on the questionnaire platform will be enabled to reduce the possibility of repeated responses from the same participant. After data collection, submitted questionnaires will also be reviewed according to prespecified data-quality criteria. The research dataset will not contain direct personal identifiers such as the child’s name, national identification number, telephone number, or detailed home address.

#### 2.4.3 Survey procedure

1. Questionnaire design and expert review (completed);
2. A pilot survey will be conducted in 1–2 kindergartens. The questionnaire will be revised if necessary based on the results (June–September 2026);
3. The final version of the questionnaire will be completed and uploaded to the Wenjuanxing platform, and the survey link and QR code will be generated (October 2026);
4. Sampling will be completed, participating kindergartens and classes will be selected, and standardized training will be provided to relevant kindergarten staff (November–December 2026);
5. Formal data collection will be conducted. After eligibility confirmation and informed consent, children will undergo standardized height and weight measurements, followed by completion of the electronic questionnaire by their primary caregivers, with assistance provided when necessary (January–June 2027);
6. After data collection, the dataset will be checked, cleaned, weighted as appropriate, and analyzed according to the prespecified statistical analysis plan (July–December 2027).

The overall study procedure and participant inclusion process are summarized in Figure 1.

**Figure 1:**
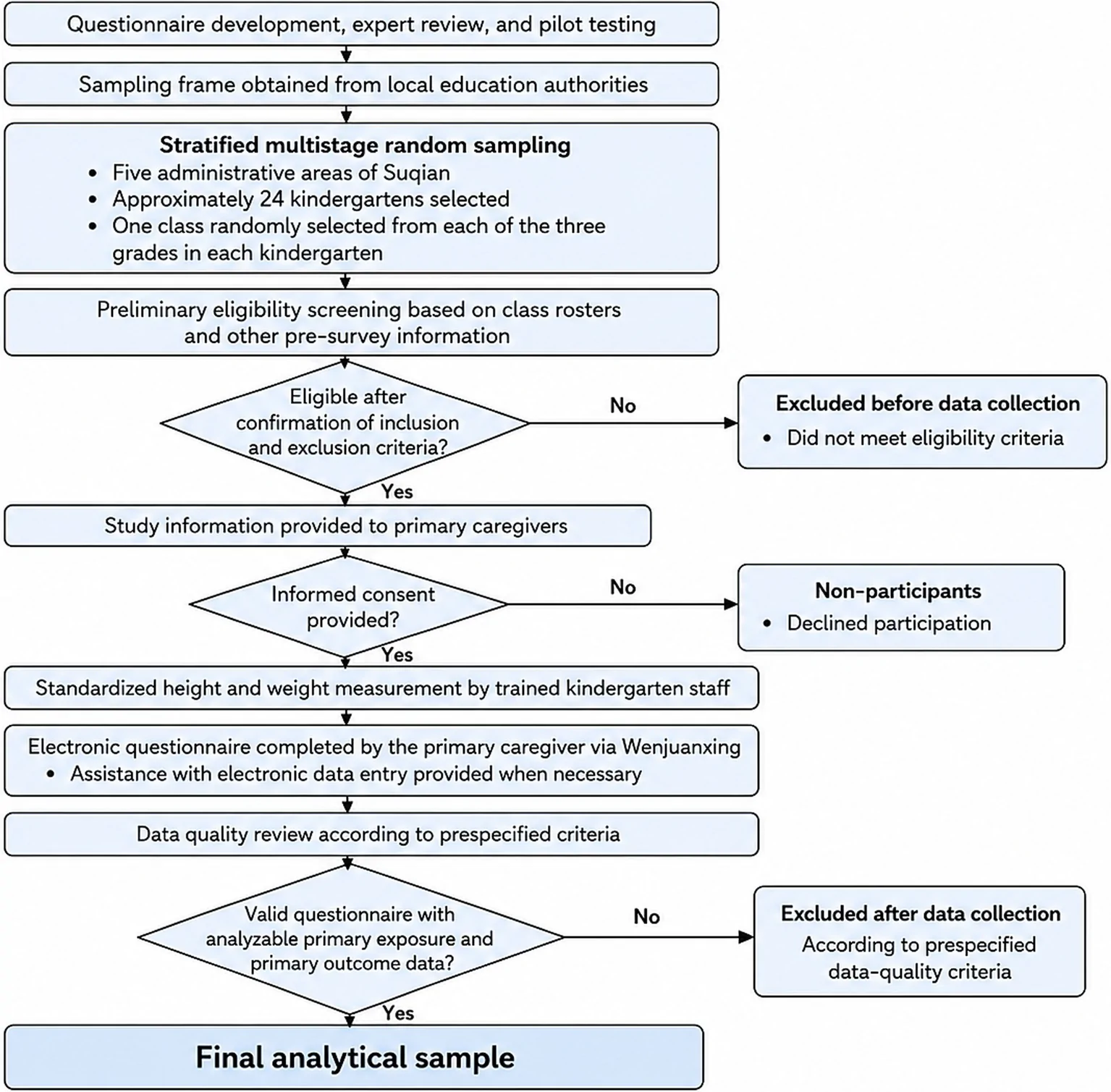
Flow chart of the study design and implementation.

### 2.5 Research instrument

Data will be collected using a structured questionnaire developed for the purposes of this study, entitled the “Questionnaire on Electronic Screen Exposure among Preschool Children” (Supplementary material 1). Questionnaire items were developed based on relevant previous studies, recommendations on children’s screen use, and child health guidelines, and were further refined according to the study objectives and characteristics of the target population. The questionnaire has undergone qualitative review by experts in child healthcare to improve the relevance, clarity, and comprehensibility of the items. As this is a study protocol, formal pilot testing and psychometric evaluation have not yet been completed; therefore, empirical estimates of content validity or test–retest reliability are not currently available.

Before formal data collection, further content validity assessment and pilot testing will be conducted. Content validity will be evaluated using structured expert ratings of the relevance and clarity of individual items, from which corresponding content validity indices will be calculated. Pilot testing will be conducted in 1–2 kindergartens that will not be included in the formal survey. The pilot study will assess item comprehensibility, appropriateness of response options, questionnaire logic, potential omissions, and completion time, and necessary revisions will be made accordingly. A subset of primary caregivers will complete the questionnaire again 7–14 days after the initial administration to assess test–retest reliability. Agreement will be assessed using kappa coefficients for categorical items and weighted kappa coefficients for ordinal items. Intraclass correlation coefficients will be used for items suitable for evaluation in continuous form, where applicable. Because the questionnaire primarily consists of independent items assessing different demographic, behavioral, and health characteristics rather than a unified scale measuring a single latent construct, an overall Cronbach’s alpha will not be calculated for the entire questionnaire.

The questionnaire covers five domains: (1) child and family characteristics, including the child’s sex and age, family caregiving structure, parental education, and household economic status; (2) screen exposure, including age at first screen exposure, weekday and weekend screen time, duration of continuous screen use, and background television exposure; (3) screen use patterns, including primary devices, content types, usage contexts, caregiver accompaniment, and parental supervision; (4) family media environment and related characteristics, including the number of electronic devices in the household, parental screen use, family rules regarding screen use, and outdoor activities; and (5) child health-related indicators, including sleep duration, sleep onset latency, bedtime resistance, and daytime sleepiness. The questionnaire also includes fields for entering the child’s height and weight. These values will be obtained from standardized measurements performed by trained kindergarten staff before questionnaire completion rather than from caregiver recall or previous records.

### 2.6 Variable definitions

1. *Screen exposure*: Screen exposure refers to the use of electronic devices with screens, including smartphones, tablets, televisions, computers, learning devices, and game consoles (22).
2. *Average daily screen time and excessive screen exposure*: Average daily screen time will be assessed using a separate questionnaire item asking the primary caregiver to report the child’s usual overall daily screen time during the past month, taking both weekdays and weekends into account. Based on relevant guidelines and previous studies in preschool children (4, 8), average daily screen time >1 h/day will be predefined as excessive screen exposure, whereas ≤1 h/day will be classified as non-excessive screen exposure and used as the reference category. Weekday and weekend screen time will also be assessed separately using prespecified duration categories to characterize screen-use patterns and for secondary analyses.
3. *Age at first screen exposure*: Age at first screen exposure refers to the age at which the child was first exposed to or began using electronic screen devices, as reported retrospectively by the primary caregiver.
4. *Background television exposure*: Background television exposure refers to situations in which the television is turned on while the child is playing, eating, or engaging in other activities, even when the child is not actively watching television (23).
5. *Body mass index and overweight/obesity*: Children’s height and weight will be measured using standardized procedures by trained kindergarten staff. Body mass index (BMI) will be calculated as weight in kilograms divided by height in meters squared (kg/m^2^). Age will be determined in months. Overweight and obesity will be classified according to the age- and sex-specific BMI reference values for Chinese children and adolescents developed by Li et al. in 2009 (24). Children meeting the criteria for either overweight or obesity will be classified as having overweight/obesity.
6. *Sleep-related indicators*: Sleep-related indicators will include total sleep duration, sleep onset latency, bedtime resistance, and daytime sleepiness (15, 25). Total sleep duration refers to the child’s average total daily sleep time over the past month, including nighttime sleep and daytime naps. Sleep onset latency refers to the time between going to bed with the intention to sleep and actually falling asleep. Bedtime resistance refers to the frequency of behaviors such as delaying bedtime, refusing to go to bed, or requiring prolonged soothing at bedtime. Daytime sleepiness refers to the frequency of tiredness, sleepiness, or difficulty waking during daytime activities. These indicators will be evaluated separately as sleep-related outcomes.

### 2.7 Data collection and quality control

#### 2.7.1 Data collection

Caregiver-reported information will be collected through the Wenjuanxing platform. The electronic questionnaire will be completed by the child’s primary caregiver based on the child’s actual situation during the past month. For caregivers who are unable to complete the electronic questionnaire independently because of limited digital skills, internet access, or device availability, trained class teachers or research staff may assist with electronic data entry based strictly on the responses provided by the caregiver.

Before questionnaire completion, children’s height and weight will be measured by trained kindergarten staff using standardized procedures. The measurement results will be communicated individually to the primary caregiver, who will enter the values into the corresponding items in the electronic questionnaire.

#### 2.7.2 Quality control

The following measures will be implemented to ensure data quality:

1. Questionnaire refinement and pilot testing
  The questionnaire used in the formal survey will be finalized after completion of the content validity assessment and pilot testing. The relevant procedures are described in Section 2.5.
2. Standardized training and instructions

Before formal data collection, standardized training will be provided to staff involved in survey implementation, assistance with electronic data entry, and anthropometric measurements. Training will cover study procedures, eligibility confirmation, questionnaire completion requirements, privacy protection, and standardized methods for measuring height and weight. The first page of the electronic questionnaire will provide standardized completion instructions and definitions of key concepts to reduce differences in interpretation.

1. Quality control of anthropometric measurements

Height and weight will be measured using equipment that meets measurement requirements, and the condition and accuracy of the equipment will be checked before formal measurements. Children will be measured without shoes and while wearing light clothing, following standardized procedures. Height will be measured to the nearest 0.1 cm and weight to the nearest 0.1 kg. Measurements that are clearly outside plausible ranges or otherwise raise concerns will be verified or repeated during the same survey session.

1. Electronic questionnaire logic control

Appropriate skip logic, plausible value ranges, and consistency checks will be incorporated into the electronic questionnaire according to the characteristics of each variable. Appropriate completeness checks will be applied to the primary exposure, primary outcome, and other key variables to reduce missing data and obvious data-entry errors. For sensitive items, such as household economic status, reasonable response options such as “prefer not to answer” will be retained.

1. Duplicate-submission control

Before formal data collection, duplicate-response restrictions available on the electronic survey platform will be enabled to reduce the possibility of repeated submissions. If more than one eligible child under the care of the same primary caregiver is selected, the caregiver may provide questionnaire information separately for each child, and trained research staff may assist with electronic entry of the additional questionnaire based on the caregiver’s responses.

1. Data-quality assessment

After data collection, records will be reviewed according to prespecified data-quality criteria. Records for which the primary exposure or primary outcome cannot be determined, or that contain serious and irreconcilable logical inconsistencies among key variables, will not be included in the corresponding primary analysis. Other partially missing data will not automatically result in exclusion of the entire questionnaire; the specific methods for handling missing data will be described in the statistical analysis section.

1. Data verification

After data export, two researchers will independently verify variable coding, value ranges, logical consistency, and implementation of the prespecified data-cleaning rules. Any identified issues will be resolved after verification, and the corresponding data-cleaning procedures will be documented.

### 2.8 Statistical analysis

All data management and statistical analyses will be performed using R software. All statistical tests will be two-sided, with *p* < 0.05 considered statistically significant. Effect estimates will be reported with 95% confidence intervals (95% CIs). Analyses will account for the stratified, multistage cluster sampling design. Survey weights will be calculated based on the actual sampling probabilities and adjusted for non-response. Survey-weighted analyses will account for both stratification and clustering.

#### 2.8.1 Descriptive analysis

Categorical and ordinal variables will be summarized as frequencies, weighted percentages, and 95% CIs. Continuous variables will be summarized as means and standard deviations or medians and interquartile ranges, as appropriate according to their distributions.

Participant demographic and family characteristics, screen exposure characteristics, family media environment, and sleep-related indicators will be described. The weighted prevalence of excessive screen exposure and overweight/obesity will be reported with 95% CIs. Weekday and weekend screen time and individual sleep-related indicators will also be described separately.

Response rates across sampling strata and kindergartens will be summarized. Where corresponding sampling-frame information is available, respondents and non-respondents will be compared with respect to known characteristics such as administrative area, kindergarten type, and grade to assess potential non-response bias.

#### 2.8.2 Primary analysis

The primary analysis will evaluate the association between excessive screen exposure and overweight/obesity. The primary exposure will be defined as average daily screen time >1 h/day, with ≤1 h/day used as the reference category. The primary outcome will be overweight/obesity.

Survey-weighted Poisson regression with a log link and robust variance estimation will be used to estimate prevalence ratios (PRs) and 95% CIs. Both unadjusted PRs and adjusted prevalence ratios (aPRs) will be reported.

The primary model will adjust for child age, sex, residential setting, parental education, monthly household income, and family living arrangement.

#### 2.8.3 Secondary analyses

Associations between screen exposure and total sleep duration, sleep onset latency, bedtime resistance, and daytime sleepiness will be examined separately as secondary analyses using survey-weighted ordinal logistic regression models.

Associations of child characteristics, family characteristics, and family media environment characteristics with excessive screen exposure will also be examined as secondary analyses. When excessive screen exposure is analyzed as a binary outcome, survey-weighted Poisson regression with robust variance estimation will be used to estimate PRs and 95% CIs.

In addition to the binary screen exposure definition used in the primary analysis, secondary analyses will examine prespecified ordered screen-time categories and weekday and weekend screen time separately to further evaluate associations between different levels of screen exposure and overweight/obesity.

#### 2.8.4 Missing data, model assessment, and sensitivity analyses

Participants for whom the primary exposure or primary outcome cannot be determined will not be included in the primary analysis. The extent and patterns of missing data will be summarized before imputation. Missing values in prespecified covariates will be handled using multiple imputation by chained equations. Estimates from the imputed datasets will be combined using Rubin’s rules. Complete-case analysis will be performed as a sensitivity analysis.

Multicollinearity among covariates will be assessed before fitting multivariable models, including evaluation using the variance inflation factor (VIF). For continuous covariates, the assumption of linearity in relation to the outcome will be assessed, and restricted cubic splines will be used where appropriate to account for nonlinear relationships.

Interactions of screen exposure with child age and sex will be evaluated as prespecified secondary effect-modification analyses. Because the primary analysis addresses a single prespecified hypothesis, no adjustment for multiple comparisons will be applied to the primary analysis. For multiple secondary and exploratory analyses, the Benjamini–Hochberg procedure will be used to control the false discovery rate.

Model diagnostics will include assessment of model convergence, relevant model assumptions, and potentially influential or outlying observations. Sensitivity analyses will include complete-case analysis, unweighted analysis, and repetition of the primary analysis using alternative screen-time categorizations to evaluate the robustness of the primary findings.

### 2.9 Ethical considerations

This study has been approved by the Ethics Committee of Suqian First Hospital (approval number: 2026-SR-0112). The study has also been registered in the Chinese Clinical Trial Registry (registration number: ChiCTR2600126288).

*Informed consent*: This study will be conducted in accordance with the ethical principles of the Declaration of Helsinki. Before any study procedures are undertaken, the child’s primary caregiver will be provided with information regarding the study objectives, study content, survey procedures, height and weight measurements, potential risks and benefits, and privacy protection. Only caregivers who voluntarily provide informed consent will proceed with the subsequent study procedures. Height and weight measurements of the children are non-invasive procedures. The full informed consent form is provided in Supplementary material 2.

*Voluntary participation*: Participation in this study is entirely voluntary. Primary caregivers may decline to participate or withdraw from the study at any time without any adverse consequences, and such decisions will not affect the child’s learning, daily activities, or any other rights or interests at the kindergarten.

*Privacy and data confidentiality*: Children’s height and weight measurements will be communicated individually to their primary caregivers. The research dataset will not contain direct personal identifiers, such as children’s names, national identification numbers, telephone numbers, or detailed home addresses. Study data will be stored securely, accessible only to authorized members of the research team, and used solely for statistical analyses and academic research related to this study.

## 3 Discussion

This study protocol aims to address the lack of evidence on screen exposure among preschool children in Suqian, a resource-limited city in eastern China. Previous studies have mainly focused on economically developed cities like Beijing and Shanghai. Evidence from regions with different socioeconomic backgrounds remains limited. An important feature of this study is that it moves beyond a single focus on total screen time and adopts a multidimensional assessment of usage patterns. It includes specific contexts such as background television exposure, screen use during meals, and screen use before bedtime. This approach allows for a more comprehensive description of children’s screen use behaviors. Digital content type may also be relevant when interpreting children’s screen-use patterns. Educational and entertainment-oriented content may differ in purpose, level of engagement, and usage context, and may therefore be differently associated with child health outcomes. The questionnaire records several common content types to provide additional context for interpreting the findings. In addition, this study incorporates a social and behavioral perspective. It considers family-related factors such as grandparental caregiving and the use of digital devices as a form of “digital nanny.” It also explores how limited caregiving resources may be associated with family media use practices. Through this design, the study is expected to provide evidence on screen exposure among preschool children in resource-limited areas. It may also offer a basis for future research and health promotion strategies.

Several methodological features are incorporated to strengthen the rigor of the study. The stratified multistage random sampling design covers all five administrative areas of Suqian and includes both public and private kindergartens. Survey-weighted analyses will account for unequal sampling probabilities, stratification, clustering, and non-response, thereby aligning the statistical analysis with the sampling design (26, 27). For the primary association between excessive screen exposure and overweight/obesity, survey-weighted Poisson regression with a log link and robust variance estimation will be used to estimate prevalence ratios directly. This approach is appropriate for cross-sectional studies with binary outcomes and avoids the potential overinterpretation of odds ratios when the outcome is not rare (28, 29). In addition, standardized anthropometric measurements and prespecified procedures for questionnaire refinement, pilot testing, and data quality control are incorporated to improve measurement consistency and data quality.

This study is also feasible in practice. Recruitment through the kindergarten system provides an efficient way to reach preschool children and their caregivers across participating sites. The use of a WeChat-based electronic questionnaire enables centralized data collection and reduces the need for manual data entry. However, web-based surveys may be affected by selective non-response and differences in digital access (30, 31). To reduce potential participation barriers, caregivers who have difficulty completing the electronic questionnaire because of limited digital skills, internet access, or device availability will be offered assistance with electronic data entry. The questionnaire is designed to take approximately 8–10 min to complete, as questionnaire length and design may influence participation and completion in web-based surveys (32). Standardized training, electronic logic checks, and direct anthropometric measurements by trained kindergarten staff are incorporated to support consistent data collection across participating sites. Sleep-related information will be collected using concise caregiver-reported indicators to reduce questionnaire burden and facilitate large-scale data collection.

This study is expected to provide context-specific epidemiological evidence on screen exposure among preschool children in a resource-limited area of eastern China. By assessing screen exposure from a multidimensional perspective, including total screen time, background television exposure, screen use during meals and before bedtime, and family media practices, the study extends beyond a single focus on total screen time. The primary analysis will provide evidence on the association between excessive screen exposure and overweight/obesity, while secondary analyses will further examine associations between screen exposure characteristics and individual sleep-related indicators. The findings may also provide a basis for future comparisons across regions with different socioeconomic contexts. From a public health perspective, the study may help identify screen-use patterns and family media characteristics that could inform future health promotion strategies for preschool children. In addition, the findings may provide baseline evidence and methodological experience for future longitudinal studies investigating the temporal relationships between screen exposure and child health outcomes.

This study also has several limitations. First, because the exposure and outcomes will be assessed at the same time, their temporal sequence cannot be established with certainty, and the observed associations should not be interpreted as causal (33). Second, screen exposure and sleep-related information will be reported by primary caregivers and may therefore be affected by recall or reporting error. Parent-reported screen use in young children may differ from objectively recorded device use (34), and parental reports of preschool children’s sleep may differ from objectively measured sleep for some sleep parameters (35). Such measurement error may lead to misclassification of screen exposure or sleep-related outcomes and may attenuate or otherwise distort the observed associations. Third, sleep will be assessed using several concise indicators rather than a validated standardized sleep scale. These indicators should therefore be interpreted as sleep-related characteristics rather than clinical sleep disorders. Fourth, although assistance will be provided to caregivers with limited digital skills, internet access, or device availability, voluntary participation through an electronic survey may still result in differential non-response. Fifth, residual confounding from unmeasured factors, including early-life characteristics and parental adiposity, cannot be completely excluded. Finally, the study will be conducted in a single city, which may limit the generalizability of the findings to populations with different geographic or socioeconomic characteristics. Future longitudinal studies incorporating objective measures of screen exposure and sleep may help clarify temporal relationships and reduce measurement error.

## 4 Conclusion

This study protocol aims to address the limited evidence on screen exposure among preschool children in resource-limited areas of eastern China. By assessing screen exposure from a multidimensional perspective, the study will provide context-specific evidence on screen-use patterns and family media characteristics. The primary analysis will examine the association between excessive screen exposure and overweight/obesity, while secondary analyses will examine associations with sleep-related indicators and characteristics associated with screen exposure. The findings may inform future health promotion strategies and provide a basis for longitudinal research on screen exposure and child health.
