# Supplementary material for: Screen exposure patterns and their associations with obesity and sleep among preschool children in a resource-limited city in eastern China: a multicenter cross-sectional study protocol
Source: Front Public Health. 2026 Sep 17;14:1901399. doi: 10.3389/fpubh.2026.1901399 (PMC13627357; doi:10.3389/fpubh.2026.1901399)
Supplement: Supplementary file 1 [file Supplementary_file_1.DOCX]

Supplementary Material 1

**Questionnaire on Electronic Screen Exposure among Preschool Children**

**Instructions for Completion**

Dear primary caregiver,

Hello! This questionnaire aims to understand the prevalence, usage patterns, and related factors of electronic screen exposure among preschool children. The research dataset will not contain direct personal identifiers, and all information will be used only for research purposes. Please complete the questionnaire based on your child’s actual situation over the past month.

This questionnaire should be completed by the child’s primary caregiver. One questionnaire should be completed for each child.

**Note:** In this questionnaire, “electronic screen” refers to devices with screens, including smartphones, tablets, televisions, computers, learning devices, and game consoles.

**Part 1. Child and Family Characteristics**

1. **What is your child’s sex?**

- 1 = Male
- 2 = Female

2. **What is your child’s age?**

_____ years _____ months

3. **Which class is your child currently enrolled in?**

- 1 = Junior class
- 2 = Middle class
- 3 = Senior class
- 4 = Preschool preparatory class / Other

4. **Is your child an only child?**

- 1 = Yes
- 2 = No

5. **How many minor siblings live with your child?**

- 0 = None
- 1 = One
- 2 = Two or more

6. **Is your child a left-behind child?**
   *Note: A left-behind child refers to a child who has been left in the care of others for ≥6 months because both parents work away from home, or one parent works away while the other is unable to provide effective care.*

- 1 = Yes
- 2 = No

7. **Who is mainly responsible for your child’s daily care?**

- 1 = Father
- 2 = Mother
- 3 = Grandparents
- 4 = Both parents
- 5 = Other relatives / Others

8. **What is the highest education level of the father?**

- 1 = Primary school or below
- 2 = Junior high school
- 3 = High school / vocational school
- 4 = College (associate degree)
- 5 = Bachelor’s degree or above
- 6 = Unknown / Not applicable

9. **What is the highest education level of the mother?**

- 1 = Primary school or below
- 2 = Junior high school
- 3 = High school / vocational school
- 4 = College (associate degree)
- 5 = Bachelor’s degree or above
- 6 = Unknown / Not applicable

10. **What was the mother’s age at childbirth?**

- 1 = <25 years
- 2 = 25–29 years
- 3 = 30–34 years
- 4 = 35–39 years
- 5 = ≥40 years
- 6 = Unknown / Not applicable

11. **What is your current family living arrangement?**

- 1 = Nuclear family (parents and child living together)
- 2 = Extended family (parents, child, and grandparents living together)
- 3 = Grandparent-led caregiving family
- 4 = Single-parent family
- 5 = Other

12. **Where does your family currently live?**

- 1 = Urban area
- 2 = Urban–rural fringe
- 3 = Town / rural area

13. **What is your approximate monthly household income?**

- 1 = <3000 RMB
- 2 = 3000–5999 RMB
- 3 = 6000–9999 RMB
- 4 = 10,000–14,999 RMB
- 5 = ≥15,000 RMB
- 6 = Prefer not to answer

14. **What type of kindergarten does your child attend?**

- 1 = Public kindergarten
- 2 = Private kindergarten
- 3 = Not sure

**Part 2. Current Status of Screen Exposure**

15. **At what age was your child first exposed to electronic screens?**

- 1 = <1 year
- 2 = 1 to <2 years
- 3 = 2 to <3 years
- 4 = 3 to <4 years
- 5 = 4 years or older
- 6 = Never or almost never exposed

16. **In the past month, taking both weekdays and weekends into account, how long did your child usually spend on electronic screens on average per day?**

- 1 = 0 minutes / almost no exposure
- 2 = <30 minutes/day
- 3 = 30 minutes to ≤1 hour/day
- 4 = >1 to <2 hours/day
- 5 = 2 to <3 hours/day
- 6 = ≥3 hours/day

17. **In the past month, how long did your child spend on electronic screens on average per day on weekdays?**

- 1 = 0 minutes / almost no exposure
- 2 = <30 minutes/day
- 3 = 30 minutes to <1 hour/day
- 4 = 1 to <2 hours/day
- 5 = 2 to <3 hours/day
- 6 = ≥3 hours/day

18. **In the past month, how long did your child spend on electronic screens on average per day on weekends or holidays?**

- 1 = 0 minutes / almost no exposure
- 2 = <30 minutes/day
- 3 = 30 minutes to <1 hour/day
- 4 = 1 to <2 hours/day
- 5 = 2 to <3 hours/day
- 6 = ≥3 hours/day

19. **In the past month, how long did your child usually use electronic screens continuously each time?**

- 1 = <15 minutes
- 2 = 15–30 minutes
- 3 = 31–60 minutes
- 4 = >60 minutes

20. **Is there a television in your child’s bedroom?**

- 1 = Yes
- 2 = No

21. **In the past month, how often was the television on in the background when your child was playing, eating, or engaging in other activities (even if the child was not actively watching)?**

- 1 = Never
- 2 = Less than 1 day per week
- 3 = 1–3 days per week
- 4 = 4–6 days per week
- 5 = Almost every day

**Part 3. Screen Use Patterns**

22. **In the past month, which electronic screen device did your child mainly use?**

- 1 = Smartphone
- 2 = Tablet
- 3 = Television
- 4 = Computer
- 5 = Learning device / reading pen
- 6 = Game console
- 7 = Other

23. **In the past month, what types of screen content did your child most often watch or use? (Multiple choices allowed)**

- 1 = Short videos (e.g., short-form video platforms)
- 2 = Long videos (e.g., cartoons, movies, children’s programs)
- 3 = Educational content
- 4 = Video games
- 5 = Video calls
- 6 = Music / children’s songs
- 7 = Other

24. **In the past month, in which situations did your child most often use electronic screens? (Multiple choices allowed)**

- 1 = Alone at home
- 2 = With parents
- 3 = With grandparents
- 4 = During meals
- 5 = Before bedtime
- 6 = During travel or waiting
- 7 = When parents are busy
- 8 = Other

25. **In the past month, who usually accompanied your child when using electronic screens?**

- 1 = Usually no one
- 2 = Father
- 3 = Mother
- 4 = Grandparents
- 5 = Both parents
- 6 = Other relatives / Others
- 7 = Not fixed

26. **In the past month, how often did your child use electronic screens during meals?**

- 1 = Never
- 2 = Less than once per week
- 3 = 1–3 times per week
- 4 = 4–6 times per week
- 5 = Almost every day

27. **In the past month, how often did your child use electronic screens within 1 hour before bedtime?**

- 1 = Never
- 2 = Less than once per week
- 3 = 1–3 times per week
- 4 = 4–6 times per week
- 5 = Almost every day

28. **In the past month, how did your child usually stop using electronic screens?**

- 1 = Stops voluntarily when the time limit is reached
- 2 = Stops after parental reminder
- 3 = Forced to stop by parents
- 4 = Often unwilling to stop / becomes upset
- 5 = Not fixed

**Part 4. Factors Related to Screen Exposure**

29. **Approximately how many electronic screen devices are available for your child at home?**

- 1 = 0–1
- 2 = 2–3
- 3 = 4–5
- 4 = ≥6

30. **Does your child have a personal electronic screen device (e.g., own smartphone, tablet, or learning device)?**

- 1 = Yes
- 2 = No

31. **In the past month, what was the father’s average daily screen time?**

- 1 = <1 hour
- 2 = 1 to <2 hours
- 3 = 2 to ≤4 hours
- 4 = >4 hours
- 5 = Unknown / Not applicable

32. **In the past month, what was the mother’s average daily screen time?**

- 1 = <1 hour
- 2 = 1 to <2 hours
- 3 = 2 to ≤4 hours
- 4 = >4 hours
- 5 = Unknown / Not applicable

33. **Are there clear rules in your family regarding your child’s screen use?**

- 1 = No clear rules
- 2 = Rules exist but are not consistently followed
- 3 = Clear rules and usually followed

34. **In the past month, how often did parents limit or guide their child’s screen use?**

- 1 = Never
- 2 = Occasionally
- 3 = About half of the time
- 4 = Most of the time
- 5 = Every time

35. **In the past month, how often did parents use electronic screens to calm the child (e.g., when crying, during meals, or when outside)?**

- 1 = Never
- 2 = Less than once per week
- 3 = 1–3 times per week
- 4 = 4–6 times per week
- 5 = Almost every day

36. **Approximately how many children’s books are available at home?**

- 1 = 0–10
- 2 = 11–30
- 3 = 31–50
- 4 = >50

37. **In the past month, how much time did your child spend on outdoor activities per day on average?**

- 1 = <30 minutes
- 2 = 30 to <60 minutes
- 3 = 1 to 2 hours
- 4 = >2 hours

**Part 5. Child Health-Related Indicators**

38. **In the past month, what was your child’s average total daily sleep duration (including nighttime sleep and naps)?**

- 1 = <8 hours
- 2 = 8 to <10 hours
- 3 = 10–13 hours
- 4 = >13 hours

39. **In the past month, how long did it usually take for your child to fall asleep after going to bed? (Sleep onset latency)**

- 1 = <15 minutes
- 2 = 15–30 minutes
- 3 = 31–60 minutes
- 4 = >60 minutes

40. **In the past month, did your child show resistance at bedtime, such as struggling, refusing to go to bed, or requiring prolonged parental soothing? (Bedtime resistance)**

- 1 = Never
- 2 = Rarely (less than once per week)
- 3 = Sometimes (1–3 times per week)
- 4 = Often (≥4 times per week)

41. **In the past month, did your child appear tired, sleepy, or difficult to wake during daytime activities (e.g., playing, eating, traveling)? (Daytime sleepiness)**

- 1 = Never
- 2 = Rarely (less than once per week)
- 3 = Sometimes (1–3 times per week)
- 4 = Often (≥4 times per week)

42. **What is your child’s height measured for this study? _____ cm**
    *Note: Please enter the height obtained from the standardized measurement conducted before questionnaire completion, to the nearest 0.1 cm.*
43. **What is your child’s weight measured for this study? _____ kg**
    *Note: Please enter the weight obtained from the standardized measurement conducted before questionnaire completion, to the nearest 0.1 kg.*
