# Supplementary material for: Screen exposure patterns and their associations with obesity and sleep among preschool children in a resource-limited city in eastern China: a multicenter cross-sectional study protocol
Source: Front Public Health. 2026 Sep 17;14:1901399. doi: 10.3389/fpubh.2026.1901399 (PMC13627357; doi:10.3389/fpubh.2026.1901399)
Supplement: Supplementary file 2 [file Supplementary_file_2.DOCX]

Supplementary Material 2

**Informed Consent Form for the Study on Electronic Screen Exposure among Preschool Children**

**Dear Primary Caregiver,**

Hello!

We are conducting a study on electronic screen exposure among preschool children. The purpose of this study is to understand the prevalence and patterns of screen exposure and to examine its associations with weight status and sleep-related indicators, as well as child, family, and family media environment characteristics associated with screen exposure. The findings may provide evidence for future child health promotion and guidance on appropriate screen use. You are invited to participate as your child’s primary caregiver.

**1. Purpose of the Study**

This study aims to investigate the prevalence and patterns of electronic screen exposure among preschool children and to examine its associations with overweight/obesity and sleep-related indicators. The study will also examine child, family, and family media environment characteristics associated with screen exposure.

**2. Participants**

This study involves preschool children aged 3–6 years and their primary caregivers. The child will undergo non-invasive height and weight measurements, and the primary caregiver will complete the electronic questionnaire. Primary caregivers may include fathers, mothers, grandparents, or other persons primarily responsible for the child’s daily care.

**3. Study Procedures**

If you agree to participate, your child’s height and weight will first be measured by trained kindergarten staff using standardized procedures. These are non-invasive measurements. The measurement results will be communicated individually to you, and you will be asked to enter the values into the electronic questionnaire. You will then complete the questionnaire based on your child’s actual situation over the past month.

The questionnaire includes the following sections:

1) Child and family characteristics;
2) Screen exposure status;
3) Screen use patterns;
4) Family-related factors associated with screen exposure;
5) Child Health-Related Indicators.

The questionnaire contains approximately 43 items and will take about 8–10 minutes to complete.

**4. Voluntary Participation**

Your participation in this study is entirely voluntary. You have the right to decide whether to participate. You may also withdraw at any time during the survey without any negative consequences. Your decision will not affect your child’s learning, daily life, or any related rights in kindergarten. You may choose not to answer any question if you prefer.

**5. Potential Risks and Discomfort**

This is an observational study involving an electronic questionnaire and non-invasive height and weight measurements. It does not involve any medication, treatment, or invasive procedures. The overall risk is minimal. The anthropometric measurements may cause brief inconvenience, and some questionnaire items concerning family circumstances, household income, or parenting practices may cause mild discomfort. You may decline to answer questions that you do not wish to answer or discontinue participation at any time.

**6. Potential Benefits**

You will not receive direct financial compensation or personal benefit from participating in this study. However, the findings may help improve understanding of screen exposure among preschool children and support the development of better guidance for healthy screen use and child health education.

**7. Privacy and Confidentiality**

The research dataset will not contain direct personal identifiers, such as your child’s name, national identification number, telephone number, or detailed home address. Your child’s height and weight measurements will be communicated individually to you. All study data will be stored securely, accessible only to authorized members of the research team, and used solely for statistical analysis and academic research related to this study. Study results may be published in academic papers or reports, but no information that directly identifies you or your child will be disclosed.

**8. Costs and Compensation**

Participation in this study is free of charge. No financial compensation will be provided.

**9. Contact Information**

If you have any questions about this study or need further information during the survey, please contact the principal investigator:

**Principal Investigator: __________
Phone: __________
Email: __________**

**10. Consent Statement**

I have read and understood the information provided above. I understand the purpose and procedures of the study, including the non-invasive measurement of my child’s height and weight and completion of the electronic questionnaire. I understand that participation is voluntary and that I may withdraw at any time without adverse consequences. I understand that the study data will be used only for research purposes.

**I have read and understood the above information, and I voluntarily agree for my child and myself to participate in this study.**

☐ I agree for my child and myself to participate
☐ I do not agree to participate
